# Prevalence and related factors of first-time suicide attempts in the past 14 days in Chinese adult patients with first-episode drug-naïve major depressive disorder

**DOI:** 10.3389/fpsyt.2024.1366475

**Published:** 2024-03-22

**Authors:** Xiaoyin Cong, Tian Zhang, Rongrong Bian, Yong Li, Junjun Liu, Xiangyang Zhang

**Affiliations:** ^1^ Department of Clinical Psychology, Jiangsu Province Hospital and The First Affiliated Hospital with Nanjing Medical University, Nanjing, China; ^2^ Department of Psychiatry, Nanjing Meishan Hospital, Nanjing, China; ^3^ Chinese Academy of Sciences (CAS) Key Laboratory of Mental Health, Institute of Psychology, Chinese Academy of Sciences, Beijing, China

**Keywords:** first-time suicide attempt, first-episode, drug-naïve, major depressive disorder, prevalence

## Abstract

**Background:**

This study aimed to identify socio-demographic, physiologic, and psychologic related factors of the first-time suicide attempt (FSA) in the past 14 days in Chinese adult patients with first-episode drug-naïve (FEDN) major depressive disorder (MDD).

**Methods:**

A total of 1718 adult patients with FEDN MDD were enrolled in this cross-sectional survey. Depression, anxiety symptoms, and suicide attempts were assessed. Additionally, biological samples were collected and measured, while Logistic regression analysis was employed to explore the risk factors for FSA in the past 14 days among FEDN MDD patients.

**Results:**

Among suicide attempters, 12.11% (208 out of 1718) reported experiencing FSA in the past 14 days. Logistic regression analysis showed that the risk factors for FSA included more severe anxiety symptoms (OR=1.37, 95%CI: 1.28-1.48, *p*<0.001), higher levels of total cholesterol (TC) (OR=1.42, 95%CI: 1.13-1.77, *p*=0.003), and elevated thyroid-stimulating hormone (TSH) (OR=1.13, 95%CI: 1.03-1.25, *p*=0.01). The regression model exhibited good discriminatory power for FSA with an area under the curve (AUC) of 0.82.

**Conclusion:**

FEDN MDD patients with more severe anxiety and higher levels of TSH and TC are more likely to develop FSA in the past 14 days. These factors are risk factors for short-term (in the past 14 days) FSA and may serve as indicators for early intervention.

## 1 Introduction

Major depressive disorder (MDD) is a mental disorder in which low mood and anhedonia persist for at least two weeks to a distressing or disabling degree (1). According to the World Health Organization (WHO) reports, MDD was one of the top 10 leading causes of disability-adjusted life years (DALYs) among people aged 10-49 in 2019 (2). Suicidal behaviors, an intentional self-inflicted act that ends in death, are one of the recognized diagnostic criteria for MDD in the DSM-5 (3). Suicide is the most severe symptom of MDD patients and may or may not be fatal (4, 5). Up to 50% of people with MDD report suicidal thoughts or suicide attempts (3, 6). People with MDD have a 20-fold higher risk of suicide attempts (7), and they are three times more likely to suicide (8).

A number of factors have been recognized as possible risk factors for suicide. According to a meta-analysis, various confounding factors such as low age, female, underweight or obesity, a longer duration of illness, and comorbid psychiatric or somatic symptoms were identified as risk factors for suicide attempts (9–11). Studies have also shown that a history of suicide attempts increases the risk of suicide, and serves as one of the strongest factors of impending suicide attempts (12–15). Therefore, it is critical to explore the first-time suicide attempt (FSA).

It is estimated that up to 13% of people with MDD who have made a non-fatal suicide attempt later die by suicide (3). 1.6% of suicide attempters die by suicide within 12 months, and 3.9% of them die by suicide within five years (16). In the first month following an emergency department visit for a psychiatric problem, 12.9% of people with MDD have attempted suicide (17). Approximately 40% of those who die by suicide have visited a physician one month before death. Of these, 20% have visited a physician one week before death (18, 19). According to a recent meta-analysis of suicide risk studies over the past 50 years, they mostly focused on predicting suicide attempts in the next 12-24 months, with less than 1% on predicting suicide attempts in the next 30 days (20). Risk factors for suicide attempts have been explored over a long term (i.e., over a lifetime or the past year), but clinicians are still frequently asked to assess the suicide risk in the near term (17).

In recent decades, researchers have attempted to identify biomarkers associated with suicide attempts, such as cholesterol levels, thyroid hormones, and impaired fasting glucose (21–23). The median time from the last psychiatric outpatient visit to the death is 18 days (24). Therefore, identifying possible biomarkers of FSA within 14 days of MDD onset is critical to prevention and treatment. In this study, different from previous studies, FSA was focused on the past 14 days.

To the best of our knowledge, this study was the first to explore FSA in the past 14 days in patients with first-episode drug-naïve (FEDN) MDD. Notably, investigating FEDN MDD patients offers distinct advantages of minimizing confounding factors such as antidepressant effects, long-term medication use, and associated medical and psychiatric complications. The objectives of this study were to (1) comprehensively analyze the occurrence and clinical characteristics of FSA in FEDN MDD patients over 14 days, and (2) identify key factors significantly associated with FSA in the past 14 days in patients with FEDN MDD.

## 2 Methods

### 2.1 Participants and settings

The protocol of this cross-sectional study was approved by the Institutional Review Board (IRB) of the First Hospital of Shanxi Medical University (ID No. 2016-Y27). All 1718 patients were consecutively recruited from the psychiatric outpatient clinic between September 2016 and December 2018, and they were all informed about this study and voluntarily signed an informed consent form.

Inclusion criteria included: (1) patients aged 18-60 years old and of Han people; (2) those who were assessed and diagnosed at enrollment by two trained and experienced clinical psychiatrists based on the diagnostic interview of the Structured Clinical Interview for DSM-IV (SCID); (3) those with first-episode depressive symptoms and a HAMD-17 score of ≥24; (4) those who never received psychotropic medication; (5) those who provided written informed consent prior to participation and were able to participate in the clinical assessment.

Exclusion criteria included: (1) patients comorbid with a serious condition, such as organic brain disease, cancer, epilepsy, brain injury, stroke, or persistent infections that required immediate medical attention (n = 9); (2) those with Axis I psychiatric disorders other than MDD, such as bipolar disorder and schizophrenia (n = 15); (3) those with substance dependence other than tobacco use (n = 9); (4) pregnant or breastfeeding women (n = 10); (5) those who refused to provide written informed consent (n = 21); and other unspecified factors (n = 14). Finally, 78 patients were excluded from the research.

### 2.2 Socio-demographic and general information

Detailed socio-demographic information including age, gender, time of onset, duration of illness, educational level and marital status was collected by questionnaire. In addition, we assessed patients’ body mass index (BMI), diastolic blood pressure (DBP) and systolic blood pressure (SBP). After at least 15 minutes of rest in a sitting posture, the right arm’s SBP and DBP were calculated by averaging two measurements with a conventional mercury sphygmometer. Each patient provided standard anthropometric measurements of their weight (kg) and height (m) to calculate their BMI using the formula: BMI = weight/(height)^2^.

### 2.3 Clinical measures

The 17-item Hamilton Rating Scale (HAMD-17) was used in the study to assess the severity of depression, with a higher score indicating more severe depressive symptoms (25). The Hamilton Anxiety Rating Scale (HAMA) was used to evaluate anxiety symptoms, with a higher score indicating more severe anxiety symptoms (26). The positive subscale of the Positive and Negative Syndrome Scale (PANSS) was used to measure psychotic symptoms. Subjects who scored more than 15 points were considered to have psychotic symptoms (27, 28). Two psychiatrists participated in pre-survey training about the HAMD, HAMA, and PANSS to ensure the reliability and consistency of scores on these scales throughout the study. Repeated assessments after the training showed interrater correlation coefficients for the total scores of the HAMD, HAMA, and PANSS > 0.8.

### 2.4 Suicide attempts

Suicide attempt is a potentially self-injurious behavior (29), which should also incorporate a high likelihood of death as well as a true intent to kill oneself (30). Information on suicide attempts was collected through face-to-face interviews, in which all participants were asked the same question: “Have you ever attempted suicide in your life?” In case of an affirmative response, they were further asked about their prior suicidal behavior, including the following questions: “When, in what way, and how many times did you attempt suicide?”. If the answers were ambiguous, the researchers would conduct additional interviews with the patient’s family members or clinicians for confirmation. Based on whether or not they had tried suicide attempts in the time frame prior to the last 14 days, we divided patients into those with and without a history of suicide. We focused on patients who had not previously attempted suicide but had their first-time suicide attempt (FSA) in the past 14 days.

### 2.5 Blood samples

Blood samples were collected from each patient between 6:00 a.m. and 8:00 a.m. following an overnight fast, and immediately sent to the Laboratory Center of the First Hospital of Shanxi Medical University before 11:00 a.m. on the same day. Then the biomarkers including total cholesterol (TC), high-density lipoproteins (HDL), triglycerides (TG), low-density lipoproteins (LDL), free tri-iodothyronine (FT3), free thyroxine (FT4), thyroid-stimulating hormone (TSH), thyroid peroxidase antibody (TPOAb), anti-thyroglobulin antibody (TgAb) and fasting blood glucose (FBG) were measured by a BECKMAN AU5800 system (Beckman Coulter, Brea, California, USA).

### 2.6 Statistical analysis

SPSS25.0 was used for statistical analysis. *p* < 0.05 was considered a statistically significant difference.

The prevalence of suicide attempts in patients with MDD was expressed as a percentage. To compare demographic and clinical factors, analysis of variance (ANOVA) and χ^2^ test were performed. Non-normally distributed rank-ordered variables were compared by the Mann-Whitney U test. On this basis, binary Logistic regression analysis was used to identify variables that had a significant effect on FSA. Bonferroni correction was used to adjust for multiple tests. A variance inflation factor (VIF) was used to measure multicollinearity among independent variables. A VIF > 5 showed multicollinearity, which was not accounted for in the final model. The multivariate model included variables in **Table 1** with P < 0.1. The discriminatory power of the regression model for FSA was determined by receiver operating characteristic (ROC) analysis. The area under the curve (AUC) was interpreted according to the classification of Hosmer et al. (0.5 = no differentiation; 0.51-0.69 = poor; 0.7-0.79 = acceptable; 0.8-0.89 = excellent; ≥0.9 = superior) (31).

**Table 1:** Socio-demographic and clinical characteristics among FEDN MDD without suicide history before the past 14 days.

	MDD without suicide history N=1580		
	Non-suicide attempts In the past 14 days N=1372	FSA in the past 14 hdays N=208	*P*
Age, years	34.55 ± 12.43	35.74 ± 12.53	0.186
Gender			0.038
male, n (%)	476(34.7%)	57(27.4%)	
female, n (%)	896(65.3%)	151(72.6%)	
Education			0.275
Junior high school, n (%)	313(22.8%)	58(27.9%)	
High school, n (%)	619(45.1%)	87(41.8%)	
University degree, n (%)	368(26.8%)	49(23.6%)	
Master’s degree, n (%)	72(5.2%)	14(6.7%)	
Marital status			0.420
Single, n (%)	407(29.7%)	56(26.9%)	
Married, n (%)	965(70.3%)	152(73.1%)	
Age of onset, years	34.35 ± 12.31	35.58 ± 12.46	0.174
Illness duration, months	6.15 ± 4.68	5.89 ± 4.26	0.728
HAMD	29.81 ± 2.75	32.22 ± 2.77	<0.001
HAMA	20.09 ± 3.08	24.31 ± 3.61	<0.001
Psychotic positive score	8.19 ± 3.38	12.76 ± 6.85	<0.001
BMI, kg/m2	24.38 ± 1.81	24.68 ± 1.76	0.025
Systolic BP, mmHg	118.24 ± 10.27	124.07 ± 11.24	<0.001
Diastolic BP, mmHg	75.29 ± 6.31	78.43 ± 7.02	<0.001
TC, mmol/L	5.12 ± 1.07	5.86 ± 1.12	<0.001
TG, mmol/L	2.14 ± 0.98	2.28 ± 1.04	0.028
HDL, mmol/L	1.24 ± 0.28	1.13 ± 0.29	<0.001
LDL, mmol/L	2.93 ± 0.84	3.21 ± 0.92	<0.001
FBG, mmol/L	5.35 ± 0.61	5.60 ± 0.76	<0.001
TSH, uIU/Ml	4.65 ± 2.29	7.08 ± 3.22	<0.001
FT3, pmol/L	4.90 ± 0.72	4.95 ± 0.74	0.545
FT4, pmol/L	16.71 ± 3.09	16.83 ± 3.24	0.628
TgAb, IU/L	20.1 (13.9, 32.5)	25.7 (16.5, 159.5)	<0.001
TPOAb, IU/L	16.4 (12.2, 29.0)	25.0 (13.6, 122.0)	<0.001

FSA, First Suicide Attempt; HAMD, Hamilton Depression Rating Scale; HAMA, Hamilton Anxiety Rating Scale; BMI, Body Mass Index; TC, Total Cholesterol; TG, Triglyceride; HDL, High-density lipoprotein cholesterol; LDL, Low-density lipoprotein cholesterol; FBG, Fasting Blood Glucose; TSH, Thyroid Stimulating Hormone; FT3, free triiodothyronine; FT4, free thyroxine; TgAb, anti-thyroglobulin; TPOAb, thyroid peroxidases antibody.

## 3 Results

A total of 1718 patients with FEDN MDD were recruited, with a mean age of (34.9 ± 12.4) years and a mean duration of illness of (6.4 ± 4.7) months, of whom 588 were males and 1130 were females. Of these, 138 had a history of suicide, and 1,580 had no history of suicide in the time frame prior to the past 14 days. The most recent suicide attempt occurred the day before the interview, while the most distant suicide attempt occurred more than a year ago. The largest number of suicide attempts was four.

### 3.1 Prevalence of suicide attempts among FEDN MDD patients with and without a history of suicide attempts

We observed that 20.1% (346/1718) of patients with FEDN MDD attempted suicide, with 13.68% (235/1718) occurring in the past 14 days, and 12.11% (208/1718) being FSA. Patients with a history of suicide attempts had higher HAMA and HAMD scores than those without a history of suicide attempts (*p* < 0.0001).

The prevalence of suicide attempts in the past 14 days was higher in patients with a history of suicide attempts (n = 27, 19.6%) than in those without a history of suicide attempts (n = 208, 13.2%) (χ^2 ^= 4.40, *p* < 0.05, OR = 1.60, 95% CI: 1.03-2.50). After controlling for HAMA and HAMD scores, FEDN MDD patients with a history of suicide attempts had an 11-fold higher risk of suicide attempts in the past 14 days than those without a history of suicide attempts (β = 2.43, Wald = 58.91, *p* < 0.0001, OR = 11.3, 95% CI: 6.09-21.00).

Compared to patients without suicide attempts, those with FSA in the past 14 days had significantly greater symptoms of depression, anxiety, and positive psychotic symptoms, as well as higher levels of blood pressure (SBP, DBP), BMI, lipids (TC, TG, HDL, LDL), thyroid hormones (TSH, TgAb, TPOAb), and FBG (*p* < 0.05). After Bonferroni correction, no significant differences were found between the groups in terms of gender, educational level, and marital status (*p* > 0.05).

### 3.2 Clinical characteristics and biochemical parameters of patients who attempted suicide in the past 14 days versus those who did not attempt suicide among those without a history of suicide attempts

Descriptive statistics of socio-demographic data and clinical variables of those who attempted suicide in the past 14 days versus those who did not attempt suicide among FEDN MDD patients without a history of suicide attempts are presented in **Table 1**. Depression, anxiety and positive symptoms were significantly greater, and blood pressure (SBP, DBP), BMI, lipids (TC, TG, HDL, LDL), thyroid hormones (TSH, TgAb, TPOAb), and FBG were all higher in patients with FSA in the past 14 days than those who did not attempt suicide (*p* < 0.05). After Bonferroni correction, there were no significant differences between the groups in terms of gender, educational level and marital status.

### 3.3 Risk factors for FSA in the past 14 days in patients with FEDN MDD

The results of binary Logistic regression analysis showed that more severe anxiety symptoms (OR = 1.37, 95% CI: 1.28-1.48, *p* < 0.001), higher levels of TC (OR = 1.42, 95% CI: 1.13-1.77, *p* = 0.003), and elevated TSH (OR = 1.13, 95% CI: 1.03-1.25, *p* = 0.010) were significant independent risk factors for FSA in the past 14 days in patients with FEDN MDD (**Table 2**).

**Table 2:** Factors associated with FSA in the past 14 days among FEDN MDD patients.

	Odds Ratio (OR)	95%CI	*P*
HAMA	1.372	1.28-1.48	<0.001
TC	1.415	1.13-1.77	0.003
TSH	1.133	1.03-1.25	0.010

FSA, First Suicide Attempt; HAMA, Hamilton Anxiety Rating Scale; TC, Total cholesterol; TSH, Thyroid Stimulating Hormone.

Adjusting for: gender, HAMA, HAMD, PANSS positive symptom, BMI, TC, TG, HDL, LDL, TSH, TgAb, TPOAb, FBG, DBP, SBP.

**Figure 1:**
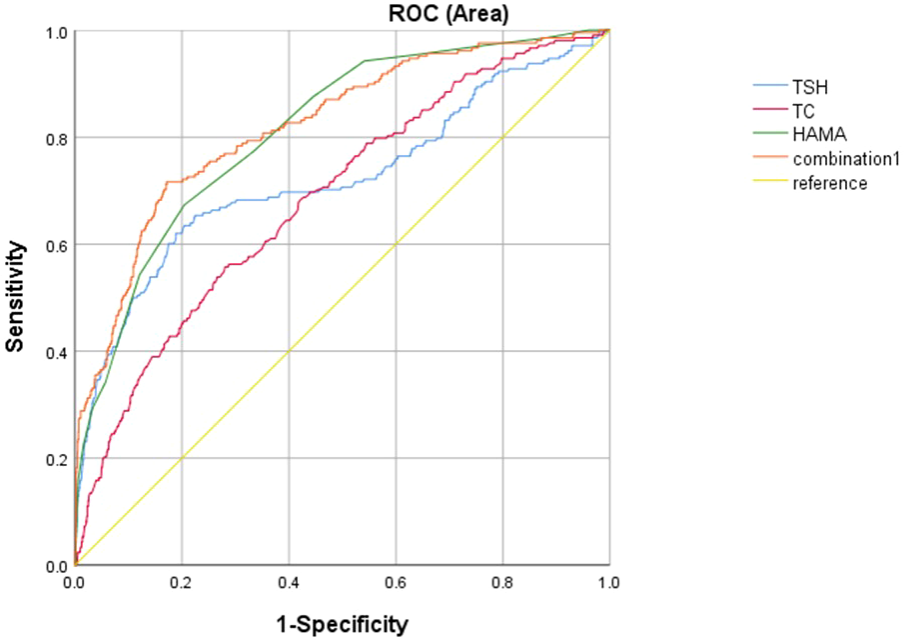
The discriminatory power of related factors for distinguishing patients with FSA in MDD in the past 14 days. The area under the curve of HAMA score, TSH, TC and the combination of these factors were 0.81, 0.72, 0.69, and 0.82, respectively. FSA, First Suicide Attempt; TSH, Thyroid Stimulating Hormone; TC, Total Cholesterol; HAMA, Hamilton Anxiety Rating Scale.

Additionally, the AUC revealed the optimal threshold values for HAMA score, TSH, and TC to differentiate FSA in the past 14 days. The optimal threshold value of the HAMA score was 22.5 (AUC: 0.81; 95% CI: 0.78-0.84), with a sensitivity of 67.3% and a specificity of 79.6%. The optimal threshold value of TSH was 6.60 (AUC: 0.72; 95% CI: 0.68-0.77), with a sensitivity and specificity of 62% and 81.1%, respectively. The optimal threshold value of TC was 5.68 (AUC: 0.69; 95% CI: 0.65-0.73), with a sensitivity and specificity of 55.8% and 71.9%, respectively. Finally, it was found that the combination of HAMA score, TC, and TSH level was the most effective in differentiating FSA in the past 14 days, with an AUC of 0.82 (95% CI: 0.79-0.85) (**Figure 1**).

## 4 Discussion

This study is the first to investigate the prevalence of FSA in the past 14 days and its associated factors in patients with FEDN MDD. Identifying individuals at risk of short-term suicide poses a significant challenge for clinical professionals and is of paramount importance. This study aimed to identify variables that differentiate between FEDN MDD patients with FSA in the past 14 days and those who did not attempt suicide. It was found that (1) 20.1% (346/1718) of FEDN MDD patients attempted suicide, of which 13.68% (235/1718) occurred in the past 14 days and 12.11% (208/1718) were FSA; (2) MDD patients with a history of suicide attempts were approximately 11 times more likely to attempt suicide in the past 14 days than those without a history of suicide attempts; (3) more severe anxiety symptoms, higher levels of TC, and TSH in patients with FEDN MDD were associated with a higher risk of FSA in the past 14 days. The combination of HAMA score, TC, and TSH level was the most effective in differentiating FSA in the past 14 days (AUC = 0.82).

The above results suggest that a 14-day window is critical. To date, only a few cross-sectional studies have reported the prevalence of short-term suicide attempts in patients with MDD. For example, Mash et al. reported that 2.6% of soldiers with MDD attempted suicide without suicidal thoughts, 16.2% of which occurred within 30 days. Among soldiers with documented suicidal thoughts, 7.4% attempted suicide, with 46.3% occurring within 30 days (32, 33). In Brazil, Brunon et al. found that 0.67% of participants had suicidal thoughts in the week prior to the interview (34). A China-based meta-analysis revealed that 20.3% of participants had attempted suicide one month before the interview (35). The prevalence of suicide attempts in previous studies varied for several reasons. First, little data are available on specific time windows related to suicidal behaviors (36). Many studies focused on suicide attempts over a long term (i.e., over a lifetime or the past year) (12). Second, there is no universally agreed upon definition of acute or chronic suicide (i.e., subsequent hours, days, weeks, or months) (12). Third, cultural and sample differences may exist. It was found that MDD patients with a history of suicide attempts were approximately 11 times more likely to attempt suicide in the last 14 days than those without a history of suicide attempts. A history of suicide attempts strongly predicts future suicide attempts or suicide deaths (37–43). According to systematic reviews and meta-analyses, adults with a history of suicide attempts have a ten-fold higher risk of suicide compared to normal people (44). MDD patients with a history of suicide attempts have a 4.84-fold higher risk of suicide than those without a history of suicide attempts (19). Sohn et al. predicted the 6-month suicide attempt rates and found that patients with a history of suicide attempts have a higher risk of suicide (OR = 2.99) (45). A history of suicide attempts was also incorporated in the final multivariate model predicting the risk of suicide in adolescents within three months (46). These studies suggest that individuals with a history of suicide attempts are more likely to attempt suicide in the long or short term. Therefore, it is critical to analyze the risk of FSA in patients with MDD.

Among MDD patients without a history of suicide attempts, the risk factors for FSA in the past 14 days were explored. It was found that more severe anxiety symptoms and higher levels of TC and TSH might contribute to short-term suicide (14 days). MDD patients with anxiety symptoms were more prone to suicide attempts, consistent with the results of several previous studies (19, 47–49). Mood disorders strongly predict future suicide attempts (50). MDD and anxiety disorders are common co-occurring psychiatric disorders (48). Patients with mild, moderate, or severe anxiety symptoms are at greater risk of suicide (49), and anxiety symptoms are more prevalent in patients with MDD who attempt suicide (3). Some researchers have suggested that the presence of depressive symptoms influences suicide attempts among patients with anxiety disorders (51). In a population-based survey, Sareen et al. conducted two follow-up assessments one year after baseline and three years after baseline, respectively. They found that a history of anxiety disorders is a unique risk factor for future suicide attempts, and MDD patients with anxiety disorders have a 4.15-fold increased risk of suicide attempts compared to those without anxiety disorders (47). In this study, anxiety remained an independent risk factor for FSA in the past 14 days in patients with MDD.

MDD patients with higher TC levels were more likely to undergo FSA in the past 14 days. In recent years, there have been an increasing number of studies on biomarkers associated with suicidal behavior, and lipid levels have emerged as a potential factor in this regard (52–55). Metabolic syndrome is common in patients with MDD, and it is associated with suicide attempts (56). One theory suggests that serum lipid levels play a crucial role in the pathogenesis of suicide; high levels of serum TC increase the risk of violent suicide (52). Higher levels of TC are associated with a higher risk of suicide in adult women (57, 58), irrespective of menopause status (59). Zhou et al. revealed that TC levels may be discovered as a potential and valuable biomarker for first-episode MDD suicide risk within two weeks (60). A recent study showed that patients with suicide attempts had higher LDL and TC but lower HDL in FEDN MDD patients (10). The underlying association mechanisms between serum cholesterol and suicide are still unclear. Surace et al. reported that higher total cholesterol blood levels may be linked to an increased risk of self-harm through the pro-inflammatory state that directly affects the brain (61). Comings et al. found that higher levels of blood cholesterol were connected to polymorphisms in the serotonin transporter gene (HTT, SLC6A4) (62), which were associated with suicidal behavior (63). Penttinen et al. hypothesized that elevated cytokine production, particularly interleukin-2 (IL-2), contributes to higher total blood cholesterol and reduced HDL cholesterol, subsequently influencing melatonin release and elevating impulsivity and suicide risk (64). Brunner et al. speculated that patients with higher cholesterol may exhibit maladaptive nutritional behaviors (such as binge eating), which was associated with suicide attempts (65).

However, different findings have been reported in other studies. For example, an association has been observed between depressive symptoms and low plasma TC levels, and low levels of TC are associated with suicide and violent death (66). Additionally, low levels of TC lead to an increased risk of suicide in patients with mental illness (67). A meta-analysis revealed that individuals with lower serum TC levels are more prone to suicidal behavior (53). A follow-up study showed that lower total cholesterol levels were significant predictors of suicide re-attempts (68). Some studies reported no association between serum cholesterol and recent suicide attempts (occurring within 72 hours before hospital admission), either violent or nonviolent (69, 70). Research including 11,653 Korean participants found that men’s depression was associated with higher triglycerides. However, there was no obvious effect of the blood lipid variables on suicidal thoughts (71). It’s important to note that drugs were used in these studies, which may have impacted the results. We recruited first-episode and drug-naïve young MDD patients to avoid the influence of many confounding factors, and this may partially explain the differences between our findings and other studies. Several other factors may account for these differences. First, different populations, genders, and ages in each study may lead to differences in findings. Cho et al. suggested that the relationship between lipids and suicide varies by age (72). Second, lipid levels fluctuate across different stages of illness. Lipid levels are lower during acute episodes of depression but increase during periods of remission (73). Some previous studies on suicide did not consider the time window, so the relationship between lipid levels and suicide appeared to vary greatly depending on the stage of MDD. Third, serum TC levels are not a reliable marker of lipid content in the brain (74). Finally, further study is needed to determine the association between TC and suicide attempts.

This study showed that elevated TSH levels in patients with MDD were associated with an increased prevalence of FSA in the past 14 days, consistent with many previous studies (75–78). A China-based study involving FEDN MDD patients found that the levels of serum TSH and TC are statistically higher in suicide attempters than in non-suicide attempters (76). Another study reported that 179 adolescents with MDD who attempted suicide had higher TSH levels, consistent with our findings, and that TSH levels are a significant predictor of suicide attempts, with an adjusted OR of 5.14 (77). Among participants with suicide attempts, there was a substantial correlation between suicide attempts and higher TSH levels (79). Furthermore, a systematic review and meta-analysis confirmed the association between higher TSH levels and suicidal behavior (75). However, some studies reported no difference in TSH levels between individuals with and without suicidal behavior (80, 81). There are several possible explanations for the inconsistency among previous studies. First, the sample sizes varied widely, ranging from 179 to 1279 subjects. Second, the sampling time was different. Some studies collected blood samples within 24 hours after suicide attempts (80), whereas some did not mention the specific time of blood collection. Therefore, future investigations are needed.

This study exhibits several strengths. First, this study used a relatively large sample size to minimize the impact of extreme data on trial results. Second, this study recruited drug-naïve MDD patients who were first-episode. Compared to previous studies, our study can relatively reduce the interference of confounding variables on FSA, such as drugs, course of disease, and comorbidities. Third, we focused on first-time suicide attempts, which made the correlation results more accurate and avoided the impact of the duration from suicide attempt to blood sampling.

However, it’s essential to acknowledge the limits of this research. First, the patients in this study were all Han people recruited from outpatient clinics; hence, it is imperative to validate our findings across diverse populations, encompassing distinct ethnicities and clinical profiles. Second, this case-control study could not determine the causal relationship between associated factors and suicide attempts in patients with MDD. Prospective cohort studies are needed to confirm our results. Third, suicide attempts were determined by interview, rather than a structured assessment tool. In addition, the data collected focused solely on suicide attempts without capturing information regarding suicidal thoughts or ideation. Despite these limitations, this study is the first to evaluate suicide attempts over a brief period of time (in the past 14 days), thereby providing valuable insights into the precise association.

## 5 Conclusion

In summary, the results of this study are essential for identifying at-risk populations for suicide attempts and making timely and appropriate clinical decisions and interventions. More severe anxiety and higher levels of TC and TSH may be risk factors for FSA in the past 14 days in patients with MDD. Future studies should focus on the changes in variables involved in the transition from no suicide risk to suicide attempts and create predictive algorithms as clinical aids to better identify patients with MDD who are at high risk of suicide. By analyzing data from a variety of sources, we hoped to provide clinicians with more valuable information for decision-making and improve prognosis. Understanding the factors contributing to FSA is critical to developing effective interventions for patients with MDD.

## Data availability statement

The raw data supporting the conclusions of this article will be made available by the authors, without undue reservation.

## Ethics statement

The protocol of this cross-sectional study was approved by the Institutional Review Board (IRB) of the First Hospital of Shanxi Medical University (ID No. 2016-Y27). The studies were conducted in accordance with the local legislation and institutional requirements. The participants provided their written informed consent to participate in this study. Written informed consent was obtained from the individual(s) for the publication of any potentially identifiable images or data included in this article.

## Author contributions

XC: Conceptualization, Data curation, Formal Analysis, Investigation, Writing – original draft, Writing – review & editing, Methodology. XZ: Resources, Supervision, Writing – review & editing. TZ: Data curation, Investigation, Writing – review & editing. RB: Data curation, Investigation, Writing – review & editing. YL: Investigation, Writing – review & editing. JL: Funding acquisition, Supervision, Writing – review & editing.
